# Newly Onset Dyspnea During Pregnancy

**DOI:** 10.1016/j.chest.2025.05.040

**Published:** 2025-10-08

**Authors:** Marie Vermant, Valerie Van Ballaer, Tine Follet, Eveline Claeys, Samuel De Bontridder, Nico De Crem, Adriana Dubbeldam, Alexandros Kalkanis, Laurens J. De Sadeleer, Ellen De Langhe, Wim A. Wuyts

**Affiliations:** a Department of Chronic Diseases and Metabolism, KU Leuven, Leuven, Belgium; b Pulmonology Department, UZ Leuven, Leuven, Belgium; c Radiology Department, UZ Leuven, Leuven, Belgium; d Pulmonology Department, AZ Sint-Lucas Gent, Ghent, Belgium; e Pulmonology Department, Regionaal Ziekenhuis Heilig Hart Leuven, Leuven, Belgium; f Department of Development and Regeneration, KU Leuven, Leuven, Belgium; g Rheumatology Department, UZ Leuven, Leuven, Belgium; h ERN ReCONNET (European Reference Network on Connective Tissue and Musculoskeletal Diseases)

A 40-year-old patient of South Asian descent with a medical history of hypertension, familial hypercholesterolemia, and obesity was experiencing dyspnea and cough and sought treatment at a regional pulmonology clinic, Heilig Hart Hospital Leuven. She had been short of breath for the last 3 years when hurrying and going uphill and had noticed a worsening of these symptoms over the previous 2 months. The cough was worse when lying on her right side. At the time of presentation, she was 24 weeks pregnant. In the months preceding her pregnancy, she had lost 9% of her body weight. Over the last 3 months, she had developed a new facial rash and pruritus on her scalp. She had sicca symptoms and felt feverish, but never took her temperature. She had previously smoked, quitting more than 5 years ago. She mentioned no exposure to mold or birds. She previously worked as a beauty specialist and was working as a housekeeper at the time of presentation. Her therapy consisted of nifedipine, 30 mg once daily, and rosuvastatin, 20 mg once daily. Clinical examination revealed the presence of bibasilar, inspiratory fine crackles. There were no clinical signs suggestive of heart failure. Bloodwork revealed moderate inflammation, with a C-reactive protein level of 13 mg/L (normal, < 5 mg/L) and an erythrocyte sedimentation rate of 52 mm/h (normal, < 20 mm/h). Pulmonary function testing (PFT) showed an FVC of 1.46 L or 42% of predicted, an FEV_1_ of 1.49 L or 52% of predicted, an FEV_1_/FVC ratio of 102%, and a total lung capacity of 2.10 L or 52% of predicted. Predictions were based on the Global Lung Function Initiative calculator. Diffusion capacity could not reliably be obtained. A thoracic radiograph (Fig 1) showed an increased reticular pattern bilaterally. The patient was referred to the Leuven University Hospital for further workup.

**Figure 1:**
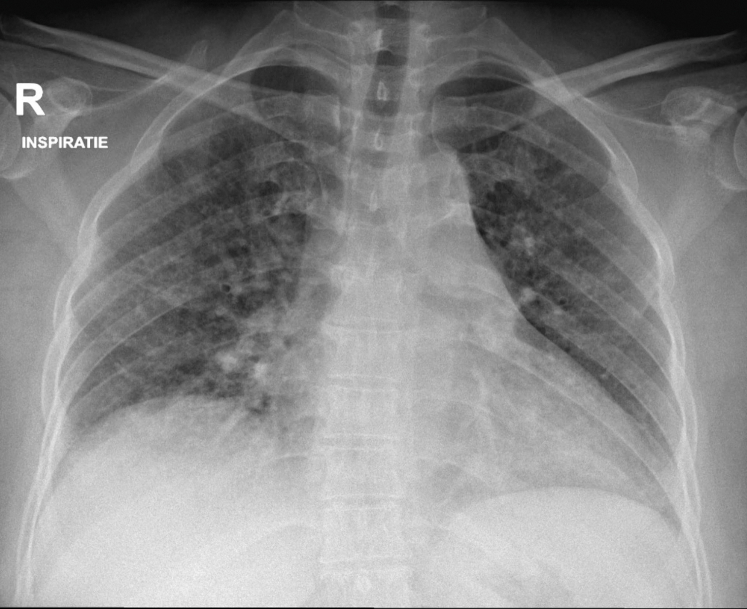
Chest radiograph on presentation.

On presentation, lung ultrasound (LUS) was performed, using a 72-window approach.^1^ The 72-window approach examines the lung via the intercostal spaces in 4 zones anteriorly and 3 zones posteriorly. The probe is placed parallel to the ribs. The anterior zones, assessed in intercostal zones 2 through 5, are the parasternal zone, the mid-clavicular zone, the anterior axillary zone, and the mid-axillary zone. The posterior zones, assessed in intercostal zones 2 through 10, are the paravertebral zone, the subscapular zone, and the posterior axillary zone.

Ethics approval for this case report was obtained via the KU Leuven/UZ Leuven ethics board (S69669).

*Question: When assessing the clips from the LUS (* Video 1 *), are there signs that point more toward an interstitial lung disease than pulmonary edema due to congestive heart failure?*

*Answer*: The ultrasound shows an extensive interstitial syndrome, with multiple B-lines in multiple zones. Pleural irregularities are present in multiple zones, supporting the diagnosis of an interstitial lung disease (ILD) more than congestive heart failure. No pleural effusion is present. Also, in Figure 2, which shows the ultrasonographic heat maps, there was no gravity-dependent pattern.

**Figure 2:**
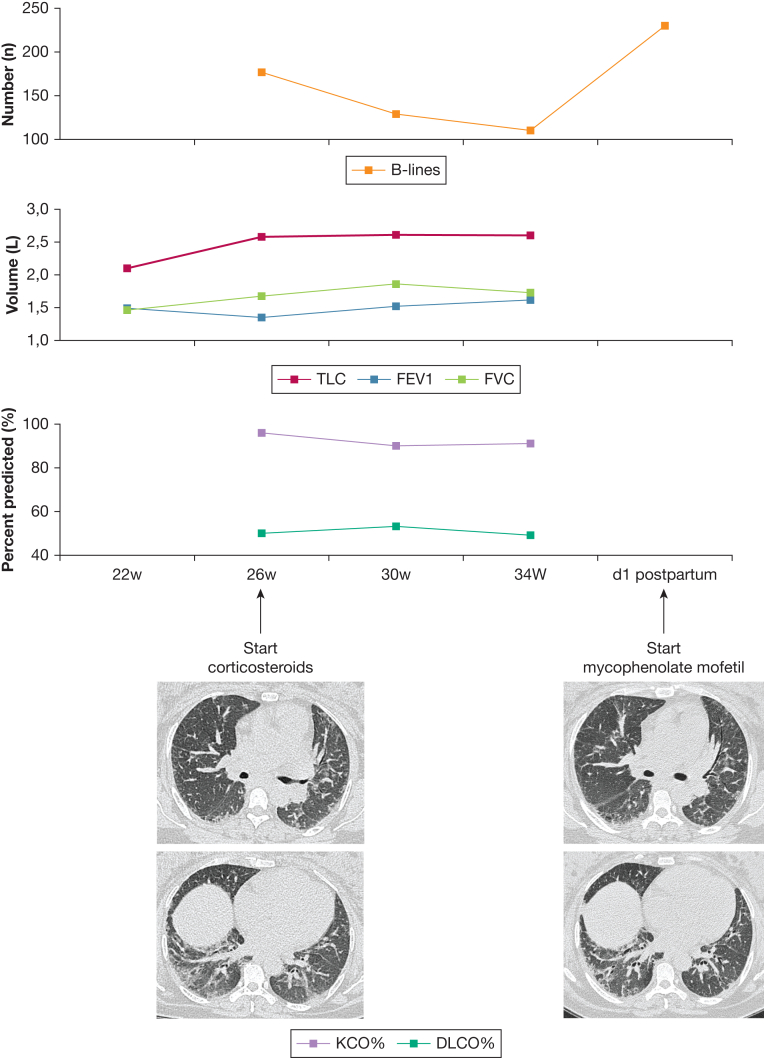
Heat maps of lung ultrasound findings at 4 different time points (26 weeks, 30 weeks, 34 weeks, and 1 day postpartum). Time is expressed as gestational age. Top: An intercostal view with 5 B-lines. Bottom: We assessed intercostal zones 2 through 5 anteriorly on the right, and intercostal zones 2 through 4 anteriorly on the left in the parasternal, mid-clavicular, anterior axillary, and mid-axillary positions. Posteriorly, we assessed intercostal zones 2 through 10 in the paravertebral, subscapular (7-10), and posterior axillary positions. All intercostal zones are colored, using a scale from 0 B-lines (green) to 10 B-lines (red). After initial improvement by administration of corticosteroids, we saw a drastic increase in the number of B-lines on day 1 postpartum compared with 34 weeks. Non-assessable intercostal spaces, due to the presence of abdominal organs, the heart, or engorged breasts in the postpartum setting, were left blank. Dlco = diffusing capacity of the lungs for carbon monoxide; Kco = carbon monoxide transfer coefficient; TLC = total lung capacity.

Using a 72-window approach, a total of 177 B-lines were counted, with a slight right-sided predominance. In addition, pleural abnormalities were present. This corresponds to an extensive interstitial syndrome. In normal pregnancy, LUS findings are similar to those of the nonpregnant population.^2^ On the basis of these findings, an urgent high-resolution CT (HRCT) scan was ordered because the pretest probability of underlying ILD in the absence of cardiac abnormalities on transthoracic echocardiography had greatly increased.

HRCT findings were suggestive of fibrotic nonspecific interstitial pneumonia. CT imaging showed the presence of bilateral triangular areas of reticulation and ground-glass attenuation, predominantly peripherally located with some extension toward the central regions, most prominent in the lower lobes, and accompanied by bilateral central tortuous traction bronchiectasis.

The indirect immunofluorescence anti-nuclear antibody test result was positive (1/1,280) with a nucleolar pattern. The connective tissue disease screen was also positive (ratio, 3.4; cutoff, ≤ 0.69) and showed a positive SSA/Ro60 titer (282 U/mL; cutoff, ≤ 6.9). Anti-NOR90 reactivity was shown (81 arbitrary units; D-tek). The rheumatoid factor assay was also positive (31.0 IU/mL; cutoff, < 3.5). Capillaroscopy showed 2 megacapillaries and 3 broadened capillaries. There were no puffy fingers, no cutaneous scleroderma, and no GI symptoms. The working diagnosis of undifferentiated connective tissue disease with an associated ILD was made.

Treatment with corticosteroids was started at an equivalent dose of 40 mg of prednisone and tapered to an equivalent dose of 10 mg by week 6. Monthly LUS and PFT were performed (Fig 3). Figure 2 shows heat maps of her ultrasonographic findings, where each intercostal zone has been scored for the number of B-lines. With high-dose corticosteroids, there was an initial improvement in her symptoms, PFT results, and ultrasonographic findings. The number of B-lines decreased in both lungs. The decrease was most pronounced in the anterior and apical zones. The initial working diagnosis was redefined as early systemic sclerosis based on the new clinical finding of a dilated blood vessel in the nail fold of her fourth digit of the left hand, in combination with the anti-NOR90 antibodies and capillaroscopy findings. Hence, the corticosteroids were kept at the low dose of 10 mg, due to the risk of scleroderma renal crisis, in addition to an increased risk of diabetes and preeclampsia. After the initial improvement and a period of stabilization, the patient’s dyspnea worsened. She was hospitalized for observation at 36 weeks gestational age. An echocardiogram did not show any abnormalities. The patient was induced at 37 weeks and delivered a healthy baby via caesarian. A postpartum CT scan showed persisting ILD, compatible with fibrotic nonspecific interstitial pneumonia. LUS showed a very extensive interstitial syndrome with a doubling of the total number of B-lines when compared with 1 month earlier (Video 2). PFT could not be performed postcaesarian. Considering her increased symptoms, the CT and ultrasound findings, mycophenolate mofetil was started at 500 mg twice daily and increased to 1,000 mg twice daily after 2 weeks.

**Figure 3:**
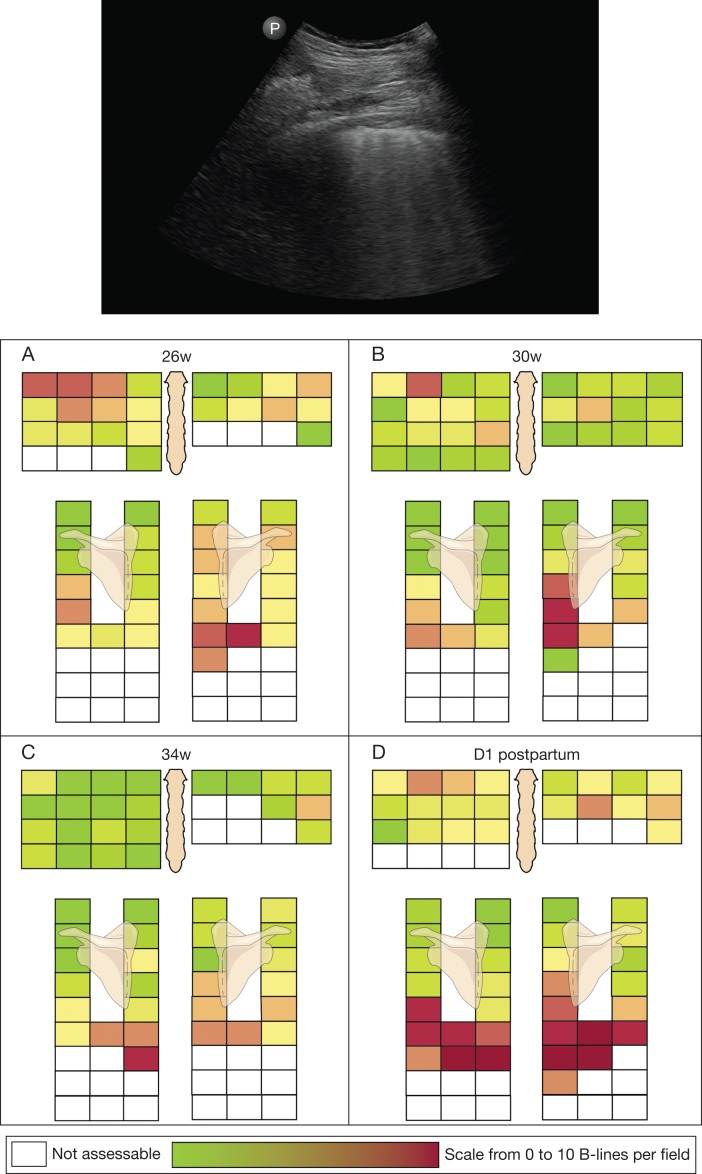
Overview of clinical evolution using HRCT imaging, pulmonary function testing, and lung ultrasound. Time is expressed as gestational age. B-lines = total number of observed B-lines; Dlco = diffusing capacity of the lungs for carbon monoxide; HRCT = high-resolution CT; Kco = carbon monoxide transfer coefficient; TLC = total lung capacity.

## Discussion

For more than 15 years, LUS has been emerging in the field of ILDs, mostly in the field of early diagnosis.^1^^,^^3^ For this patient, we used ultrasound to guide our clinical decision-making, providing clues in the initial diagnosis and follow up of this new interstitial lung disease. The current literature focuses on the detection of interstitial lung disease in systemic autoimmune rheumatic diseases, mostly in the area of early diagnosis.^4^, ^5^, ^6^, ^7^, ^8^, ^9^ There is no consensus yet on the preferred protocol for ultrasound, and multiple options have been investigated. The published methods differ in the positioning of the ultrasound probe in relation to the ribs and the number of zones scanned. The probe can be placed parallel or perpendicular to the ribs. The number of scanned zones varies from 14 zones to 72 zones.^4^^,^^7^, ^8^, ^9^, ^10^ In this patient, we opted to use the parallel 72-window approach, to gain as much information as possible. The cutoff of the total number of B-lines also varies, but most studies state that fewer than five B-lines is normal. The cutoff used in SARD-ILD differs from the B+ line described in the BLUE protocol for acute respiratory failure, which is defined by three or more B-lines in a single view.^11^

To the best of our knowledge, there are no reports on the use of ultrasound in the follow-up of ILDs after treatment initiation. In this patient, the number of B-lines decreased while her functional vital capacity and gas transfer (diffusing capacity of the lungs for carbon monoxide) increased with treatment. When her symptoms greatly increased at the end of pregnancy, we noticed that the number of B-lines doubled, and the postpartum CT scan showed extensive ground-glass opacities. We think this case report could be an initial step to promote further research, not only in the early detection of ILD, but also in the assessment of the effectiveness of inflammatory treatment by LUS in addition to PFT.

To conclude, LUS is a highly investigated tool in systemic autoimmune rheumatic diseases-ILD, with initial studies focusing on systemic sclerosis and, more recently, rheumatoid arthritis. LUS played a crucial role in this case as it provided us with critical information concerning the probability of an underlying ILD in this pregnant patient. Furthermore, this case shows the feasibility of using LUS as a means of follow-up for a pregnant patient with an underlying ILD, in addition to PFT, changes in symptoms, and clinical examination.

## Reverberations

1. *LUS in pregnant patients with respiratory symptoms is feasible*.
2. *LUS can play an essential role in the detection of interstitial lung disease in patients in whom radiation is to be maximally avoided*.
3. *LUS could potentially be used for follow-up of antiinflammatory medication, although further research is warranted*.

## Funding/Support

M. V. receives funding from Research Foundation Flanders 1SE4324N.

## Financial/Nonfinancial Disclosures

The authors have reported to *CHEST* the following: The institution at which M. V. works has received payments for educational purposes from Boehringer Ingelheim and conference support from Sanofi. E. D. L. has received consulting fees from Amgen, Argenx, and GSK, honoraria from Actelion and Lilly, and conference support from Pfizer, Boehringer Ingelheim, Lilly, and MSD. E. D. L. has been on data safety monitoring or advisory boards of AC Immune, Boehringer Ingelheim, Amgen, AstraZeneca, GSK, Novartis, and Otsuka. E. D. L. is co-president of the Working Group Rare Diseases, Belgian Royal Society of Rheumatology. All payments have been made to the institution at which E. D. L. works. W. A. W. has received grants from Roche, Boehringer Ingelheim, Galapagos, and Alentis, all paid to the institution at which W. A. W. works. None declared (V. V. B., T. F., E. C., S. D. B., N. D. C., A. D., A. K., L. J. D. S.).

## Supplementary Data

Video 1 Lung *ultrasound* on presentation. *Clip showing a selection of 3-second recordings (10 of 72 intercostal zones, anterior and posterior chest, right and left hemithorax). AA = anterior axillary; L = left; MA = mid-axillary; MC = mid-clavicular zone; PA = posterior axillary; PS = parasternal zone; PV = paravertebral; R = right*.

Video 2 Lung *u*ltrasound postpartum. *Clip showing a selection of 3-second recordings (5 of 72 intercostal zones, anterior and posterior chest, right and left hemithorax). AA = anterior axillary; L = left; PS = parasternal zone; PV = paravertebral; R = right*.
